# Clinical and HLA genotype analysis of immune checkpoint inhibitor-associated diabetes mellitus: a single-center case series from China

**DOI:** 10.3389/fimmu.2023.1164120

**Published:** 2023-06-09

**Authors:** Yi-chen Liu, Hong Liu, Shao-li Zhao, Ke Chen, Ping Jin

**Affiliations:** Department of Endocrinology, The Third Xiangya Hospital, Central South University, Changsha, Hunan, China

**Keywords:** immune checkpoint inhibitors, diabetes mellitus, HLA genotypes, fulminant type 1 diabetes, type 1 diabetes

## Abstract

**Objective:**

To investigate the clinical characteristics and HLA genotypes of patients with immune checkpoint inhibitor-associated diabetes mellitus (ICI-DM) in China.

**Methods:**

We enrolled 23 patients with ICI-DM and 51 patients with type 1 diabetes (T1D). Clinical characteristics of the patients were collected. HLA-DRB1, HLA-DQA1, and HLA-DQB1 genotyping was conducted via next-generation sequencing.

**Results:**

The ICI-DM patients had a male predominance (70.6%), a mean body mass index (BMI) of 21.2 ± 3.5 kg/m^2^, and a mean onset of ICI-DM in 5 (IQR, 3-9) cycles after ICI therapy. Most (78.3%) ICI-DM patients were treated with anti-PD-1, 78.3% presented with diabetic ketoacidosis, and all had low C-peptide levels and received multiple insulin injections. Compared to T1D patients, ICI-DM patients were significantly older (57.2 ± 12.4 *vs* 34.1 ± 15.7 years) and had higher blood glucose but lower HbA1c levels (*P*<0.05). Only two (8.7%) ICI-DM patients were positive for islet autoantibodies, which was lower than that in T1D patients (66.7%, P<0.001). A total of 59.1% (13/22) of ICI-DM patients were heterozygous for an HLA T1D risk haplotype, and DRB1*0901-DQA1*03-DQB1*0303 (DR9) and DRB1*0405-DQA1*03-DQB1*0401 were the major susceptible haplotypes. Compared to T1D, the susceptible DR3-DQA1*0501-DQB1*0201 (DR3) and DR9 haplotypes were less frequent (17.7% *vs* 2.3%; *P*=0.011 and 34.4% *vs* 15.9%; *P*=0.025), whereas the protective haplotypes (DRB1*1101-DQA1*05-DQB1*0301 and DRB1*1202-DQA1*0601-DQB1*0301) were more frequent in ICI-DM patients (2.1% *vs* 13.6%; *P*=0.006 and 4.2% *vs* 15.9%; *P*=0.017). None of the ICI-DM patients had T1D-associated high-risk genotypes DR3/DR3, DR3/DR9, and DR9/DR9. Among the 23 ICI-DM patients, 7 (30.4%) presented with ICI-associated fulminant type 1 diabetes (IFD), and 16 (69.6%) presented with ICI-associated type 1 diabetes (IT1D). Compared to IT1D patients, IFD patients exhibited marked hyperglycemia and low C-peptide and HbA1c levels (*P*<0.05). Up to 66.7% (4/6) of IFD patients were heterozygous for reported fulminant type 1 diabetes susceptibility HLA haplotypes (DRB1*0405-DQB1*0401 or DRB1*0901-DQB1*0303).

**Conclusion:**

ICI-DM shares similar clinical features with T1D, such as acute onset, poor islet function and insulin dependence. However, the lack of islet autoantibodies, the low frequencies of T1D susceptibility and high frequencies of protective HLA haplotypes indicate that ICI-DM represents a new model distinct from classical T1D.

## Introduction

Immune checkpoint inhibitors (ICIs), which can regulate T-cell activity and restore the host’s immune response against cancer, are being increasingly used for the treatment of solid tumors (1). ICIs usually include antibodies against programmed cell death-1 (PD-1) or its ligand (PD-L1) or cytotoxic T lymphocyte associated antigen 4 (CTLA-4) (2, 3). The inhibitory costimulatory molecules play a key role in regulating peripheral tolerance; thus, checkpoint blockade may also lead to immune-related adverse events (irAEs). Autoimmune endocrinopathies are the most common irAEs and may involve the pituitary, adrenal glands, thyroid and pancreas (4). Immune checkpoint inhibitor-associated diabetes mellitus (ICI-DM) is a rare but often life-threatening side effect (5–7). A retrospective analysis based on the US Food and Drug Administration (FDA) adverse event reporting system showed that the incidence of ICI-DM was 1.27% (8). Kotwal (6) et al. analyzed the data of 1444 cancer patients treated with CTLA-4 inhibitors or PD-1 inhibitors at the Mayo Clinic and found that the incidence of ICI-DM was 1.4%. ICI-DM is characterized by acute onset of hyperglycemia and insulin deficiency, which share analogous clinical characteristics with type 1 diabetes (T1D). As reported, there are two subtypes of ICI-DM, ICI-associated fulminant type 1 diabetes (IFD) and ICI-associated type 1 diabetes (IT1D) (9–14).

Although there has been a growing number of ICI-DM cases with the wide use of ICIs, the pathogenesis of ICI-DM remains unclear. It is well known that human leukocyte antigen (HLA) class II genes, especially HLA-DRB1-DQA1-DQB1, confer the highest risk for T1D (15), and susceptible HLA-DRB1-DQA1 genes vary among different ethnicities. In the Caucasian population, T1D is strongly associated with HLA-DR3-DQA1*0501-DQB1*0201 (DR3) and DR4-DQA1*0301-DQB1*0302 (DR4) haplotypes, whereas HLA DR3/DR4 are the highest risk genotype (16). In contrast, the major susceptible haplotypes in Japanese and Korean T1D are DR3, DR4 and DRB1*0901-DQA1*0302-DQB1*0303 (DR9) (17, 18), whereas HLA DR3/DR9, DR3/DR3 and DR9/DR9 are high-risk T1D genotypes in Chinese individuals (19). Given that ICI-DM has some clinical features similar to T1D, it is speculated that HLA genes are also associated with ICI-DM susceptibility. However, the impact of HLA genes on ICI-DM susceptibility has rarely been reported, and most of the data were from Caucasian populations (20–22). Herein, we describe the clinical characteristics and HLA genotypes of patients with ICI-DM of Chinese origin. To our knowledge, this is the largest sample size of ICI-DM patients in China.

## Materials and methods

### Ethics statement

This study was approved by the Institutional Ethics Committee of the Third Xiangya Hospital (NO. I22169). Written informed consent was obtained from all subjects in this study. All procedures were performed according to the World Medical Association’s Declaration of Helsinki.

### Subjects

Between January 2021 and December 2022, 23 patients with ICI-DM (6 female and 17 male) and 51 new-onset T1D patients (21 female and 30 male) were recruited from the Third Xiangya Hospital of Central South University in succession. Patients with ICI-DM who met the following criteria were included: 1) new diagnosis of insulin-dependent diabetes after exposure to ICIs and 2) no history of diabetes before the use of ICIs. T1D patients were included by the following criteria: 1) diagnosis of diabetes; 2) acute onset and presence of diabetic ketosis or ketoacidosis; and 3) insulin dependency at the time of diagnosis. Fulminant type 1 diabetes (FT1D) met the following diagnostic criteria (23): 1) diabetic ketosis or ketoacidosis occurring soon after the onset of hyperglycemic symptoms; 2) plasma glucose ≥16.0 mmol/L and HbA1c <8.7% at the first visit; and 3) fasting serum C-peptide level <0.10 nmol/L or postprandial serum C-peptide <0.17 nmol/L at onset. Immune checkpoint inhibitor-associated fulminant type 1 diabetes (IFD) was defined as fulminant type 1 diabetes induced by exposure to ICIs. Immune checkpoint inhibitor-associated type 1 diabetes (IT1D) is defined as type 1 diabetes induced by exposure to ICIs (10). ICI-DM included IFD and IT1D.

### Biochemical assays

The serum levels of blood glucose, C-peptide, sex hormone, growth hormone, thyroid hormone, cortisol, adrenocorticotropic hormone (ACTH), thyroid peroxidase antibody (TPOAb), thyroglobulin antibodies (TGAb), and thyrotrophin receptor antibody (TRAb) were all detected by electrochemiluminescence immunoassay (Roche Cobas 6000 automatic electrochemiluminescence analyzer). Glycosylated hemoglobin was measured by high-performance liquid chromatography (HA8180 automatic glycosylated hemoglobin analyzer).

### Iselet autoantibody assays

Glutamate decarboxylase antibody (GADA), protein tyrosine phosphatase autoantibody (IA−2A) and zinc transporter 8 autoantibody (ZnT8A) were detected by radioligand assays (RLAs) with a respective sensitivity and specificity of 82% and 97.8% for GADA, 76% and 100% for IA-2A, and 72% and 100% for ZnT8A according to the 2016 islet autoantibody standardization program (IASP 2016) (24). The presence of IAA was determined by the electrochemiluminescence (ECL) method, with a sensitivity of 52% and specificity of 100%, according to the IASP 2020 (25).

### HLA genotype

The genomic DNA of human blood was extracted with the American OMEGA Company kit and stored at -80 °C after concentration determination. HLA-DRB1, HLA-DQA1, and HLA-DQB1 genotyping was conducted via next-generation sequencing (NGS) by Genesky Biotechnologies Inc. in Shanghai. Multiplex PCR amplifications of the HLA loci, quality control and filtering, and data processing were performed as previously described (26). The HLA genotype was obtained by designing specific primers for specific HLA loci to amplify gene fragments and combining them with a large amount of data obtained by high-throughput sequencing.

### Statistical analysis

Data are shown as the mean ± standard deviation (SD) or proportion (%), and continuous variables that were not normally distributed are described as the median and interquartile range (IQR). Comparison of groups was carried out with independent sample *t* tests, and comparison of categorical variables employed the chi-square test. Statistical analyses were performed using GraphPad Prism 8.0. All tests were two-tailed, and a probability value of *P* < 0.05 was considered statistically significant. Owing to the number of statistical tests we performed, a Bonferroni correction for multiple testing was applied. If two independent comparisons were tested, the significance level p<0.05 was divided by two, which provides a significance level corrected for multiple testing: p<0.025.

## Result

### Comparison of clinical characteristics between ICI-DM and T1D patients

The clinical characteristics of ICI-DM and T1D patients are shown in **Table 1**. Among the 23 ICI-DM patients, 43.5% had lung cancer, 8.7% had tongue carcinoma, 8.7% had gastric carcinoma, and the remaining were affected by different types of carcinomas. Most of the ICI-DM patients received monotherapy: 18 (78.3%) patients were treated with anti-PD-1 (5 camrelizumab, 6 pembrolizumab, 4 sintilimab, 2 tislelizumab, and 1 penpulimab), 4 (17.4%) patients were treated with anti-PD-L1 (2 durvalumab and 2 atezolizumab), and one patient received cadonilimab (PD-1/CTLA-4 bispecific antibody). The ICI-DM patients had a male predominance (70.6%), a mean body mass index (BMI) of 21.2 ± 3.5 kg/m^2^, and a mean onset of ICI-DM in 5 (IQR, 3-9) cycles after ICI therapy. At diagnosis, 78.3% of ICI-DM patients had diabetic ketoacidosis, 34.8% of patients had at least one other endocrine irAE, and autoimmune thyroid disease was the most common (26.1%). All ICI-DM patients had very low C-peptide levels and received multiple insulin injections.

**Table 1:** Clinical characteristics of ICI-DM, IFD, IT1D and T1D patients.

	ICI-DM			T1D (n=51)	ICI-DM *vs* T1D		IFD *vs* IT1D	
	Total (n = 23)	IFD (n=7)	IT1D (n=16)		t/χ^2^	*p value*	t/χ^2^	*p value*
Age (years)	57.2 ± 12.1*	53.7 ± 15.9	58.8 ± 10.3	33.8 ± 15.3	6.489	<0.001	0.915	0.371
Gender (female/male)	6/17	3/4	3/13	21/30	1.557	0.298	1.468	0.226
BMI (kg/m2)	21.2 ± 3.5	21.7 ± 4.6	21.0 ± 3.0	21.2 ± 3.3	0.008	0.993	0.462	0.649
Time to ICI-DM onset (cycle)	5 (3-9)	5 (2-9)	5 (3-9)	/		/	0.488	0.630
Diabetic ketoacidosis (%)	18/23 (78.3)	6/7 (85.7)	12/16 (75.0)	34/51 (66.7)	1.021	0.313	0.329	0.567
Diabetes symptoms (%)	19/23 (82.6)	6/7 (85.7)	13/16 (81.3)	41/51 (80.4)	0.051	0.822	0.068	0.795
BG (mmol/L)	19.6 ± 11.2*	30.7 ± 15.3#	14.7 ± 2.8	11.2 ± 3.5	4.861	<0.001	4.121	<0.001
C-peptide(ng/ml)	0.34 ± 0.38	0.09 ± 0.12	0.45 ± 0.40	0.40 ± 0.45	0.575	0.567	2.262	0.034
HbA1c (%)	8.9 ± 1.9*	7.6 ± 0.8#	9.5 ± 1.9	10.6 ± 2.5	2.813	0.006	2.514	0.020
GADA (%)	2/23 (8.7)*	1/7 (14.3)	1/16 (6.3)	27/51 (52.9)	13.02	<0.001	0.396	0.529
ICA (%)	2/23 (8.7)*	1/7 (14.3)	1/16 (6.3)	21/51 (41.2)	7.807	0.005	0.396	0.529
IAA (%)	0	0	0	9/51 (17.6)	4.621	0.032	/	/
IA-2A (%)	0*	0	0	11/51 (21.6)	5.827	0.016	/	/
ZnT8A (%)	0	0	0	8/51 (15.7)	4.045	0.044	/	/

ICI-DM, immune checkpoint inhibitor-associated diabetes mellitus; IFD, immune checkpoint inhibitor-associated fulminant type 1 diabetes; IT1D, immune checkpoint inhibitor-associated type 1 diabetes; T1D, type 1 diabetes; GADA, glutamic acid decarboxylase antibody; ICA, islet cell antibody; IAA, insulin autoantibody; IA-2A, protein tyrosine phosphatase autoantibody; ZnT8A, zinc transporter 8 autoantibody. P value <0.025 was considered significant, as we had to correct our analysis for multiple testing (P value of 0.025 was calculated as 0.05 divided by 2). *P <0.025 ICI-DM vs T1D; #P<0.025 IFD vs IT1D.

Compared to T1D patients, ICI-DM patients were significantly older (57.2 ± 12.4 *vs* 34.1 ± 15.7 years, *P*<0.001) and had higher blood glucose (19.6 ± 11.2 *vs* 11.2 ± 3.5 mmol/L, *P*<0.001) but lower HbA1c levels (8.9 ± 1.9% *vs* 10.6 ± 2.5%, *P*=0.006, **Table 1**). No significant discrepancies were observed in sex, BMI, DKA, or C-peptide level between ICI-DM and T1D patients. The frequencies of GADA and ICA in ICI-DM patients were significantly lower than those in T1D patients (8.7% *vs* 52.9% and 8.7% *vs* 41.7%, *P*<0.001 and *P*=0.005). No ICI-DM patients were positive for IAA, IA-2 or ZnT8A. No patient who was negative for islet antibody at diagnosis developed antibodies in the 2-year follow-up.

### Comparison of clinical characteristics between IFD and T1D

Among the 23 ICI-DM patients, 7 (30.4%) met the criteria for IFD, and 16 (69.6%) met the criteria for IT1D. The clinical characteristics of IFD and T1D are shown in **Table 1**. Compared to IT1D patients, IFD patients exhibited marked hyperglycemia (14.7±2.8 vs 30.7±15.3 mmol/L, P<0.001), low HbA1c levels (7.6 ± 0.8% vs 9.5 ± 1.9%, P=0.020) and low C-peptide (0.09 ± 0.12 vs 0.45 ± 0.40 ng/ml, P=0.034). No significant discrepancies were observed in age, sex, BMI, DKA, time to diagnosis in cycles and islet autoantibodies between IFD and IT1D.

### HLA-DRB1-DQA1-DQB1 haplotypes in ICI-DM and T1D

According to a previous report in a Chinese population (19), HLA DR3, DR4, DR9, DRB1*0901-DQA1*05-DQB1*0201, DRB1*0301-DQA1*03-DQB1*0303, and DRB1* 0405-DQA1*03-DQB1*0401 were categorized as T1D susceptible haplotypes, while DRB1*1101-DQA1*05-DQB1*0301, DRB1*0803-DQA1*0103-DQB1*0601, DRB1*1202-DQA1*0601-DQB1*0301, DRB1*1501-DQA1*0102-DQB1*0602 and DRB1*1401-DQA1*0101-DQB1*0503 were recognized as protective haplotypes.

In total, HLA genotyping was performed in 95.7% (22/23) of ICI-DM and 94.1% (48/51) of T1D patients. As shown in **Table 2**, DR9 and DRB1*0405-DQA1*03-DQB1*0401 are the major T1DM susceptible haplotypes, whereas DRB1*1101-DQA1*05-DQB1*0301 and DRB1*1202-DQA1*0601-DQB1*0301 are the major T1DM protective haplotypes in ICI-DM. The frequencies of T1D susceptible haplotypes DR3 and DR9 in ICI-DM patients were lower than those in T1D patients (2.3% *vs* 17.7%; *P*=0.011 and 15.9% *vs* 34.4%; *P*=0.025), whereas the frequencies of T1D protective haplotypes DRB1*1101-DQA1*05-DQB1*0301 and DRB1*1202-DQA1*0601-DQB1*0301 were significantly higher in ICI-DM patients (13.6% *vs* 2.1%; *P*=0.006 and 15.9% *vs* 4.2%; *P*=0.017). A total of 59.1% (13/22) of ICI-DM patients were heterozygous for a susceptible HLA haplotype, 22.7% had both susceptible and protective haplotypes, and 31.8% carried only protective HLA haplotypes (**Table 3**). The frequency of total susceptible haplotypes in ICI-DM patients was significantly lower than that in T1D patients (59.1% *vs* 83.3%, *P*=0.038). However, the frequency of total protective haplotypes was significantly higher in ICI-DM patients than in T1D patients (54.5% *vs* 22.9%, *P =*0.014, **Table 3**).

**Table 2:** HLA-DRB1-DQA1-DQB1 haplotype frequencies in ICI-DM, IFD, IT1D and T1D patients.

DR-DQ Haplotypes	ICI-DM			T1D (n= 96)	ICI-DM *vs* T1D		IFD *vs* IT1D
	Total (n = 44)	IFD (n=12)	IT1D (n=32)		OR(95%CI)	*p value*	*p value*
Susceptible Haplotypes							
DR3(%)	1/44 (2.3)*	0(0.0)	1/32 (3.1)	17/96 (17.7)	0.11 (0.01-0.68)	0.011	0.536
DR4(%)	1/44 (2.3)	0(0.0)	1/32 (3.1)	2/96 (2.1)		0.943	0.536
DR9(%)	7/44 (15.9)	3/12 (25.0)	4/32 (12.5)	33/96 (34.4)	0.47 (0.23-0.92)	0.025	0.312
DRB1*0301-DQA1*03-DQB1*0303(%)	0 (0.0)	0 (0.0)	0 (0.0)	2/96 (2.1)		0.667	/
DRB1*0405-DQA1*03-DQB1*0401(%)	4/44 (9.1)	1/12(8.3)	3/32 (9.4)	10/96 (10.4)		0.808	0.915
DRB1*0901-DQA1*05-DQB1*0201*%)	0 (0.0)	0 (0.0)	0 (0.0)	2/96 (2.1)		0.943	/
Protective Haplotypes							
DRB1*1101-DQA1*05-DQB1*0301(%)	6/44 (13.6)*	1/12 (8.3)	5/32 (15.6)	2/96 (2.1)	2.61 (1.36-3.83)	0.006	0.530
DRB1*1202-DQA1*0601-DQB1*0301(%)	7/44 (15.9)*	0 (0.0)	7/32 (21.9)	4/96 (4.2)	2.22 (1.17-3.42)	0.017	0.259
DRB1*1401-DQA1*0101-DQB1*0503(%)	1/44 (2.3)	1/12 (8.3)	0 (0.0)	0 (0.0)		0.138	0.098
DRB1*0803-DQA1*0103-DQB1*0601(%)	1/44 (2.3)	0 (0.0)	1/32 (3.1)	3/96 (3.1)		0.779	0.536
DRB1*1501-DQA1*0102-DQB1*0602(%)	1/44 (2.3)	0 (0.0)	1/32 (3.1)	4/96 (4.2)		0.575	0.536

Data are presented as n (%). Abbreviations: DR3, DRB1*0301-DQA1*05-DQB1*0201; DR4, DRB1*0405-DQA1*03-DQB1*0302; DR9, DRB1*0901-DQA1*03-DQB1*0303; NS, no significance. P value <0.025 was considered significant, as we had to correct our analysis for multiple testing (P value of 0.025 was calculated as 0.05 divided by 2). *P <0.025 ICI-DM vs T1D.

**Table 3:** HLA susceptible and protective haplotype analysis in ICI-DM and T1D patients.

HLA Haplotypes	ICI-DM	T1D	χ^2^	*p value*
	(n = 22)	(n = 48)		
Susceptible only (%)	8/22 (36.4)*	34/48 (70.8)	7.468	0.006
Susceptible and protective (%)	5/22 (22.7)	6/48 (12.5)	1.191	0.275
Neutral (%)	2/22 (9.1)	3/48 (6.3)	0.184	0.668
Protective only (%)	7/22 (31.8)*	5/48 (10.4)	4.865	0.027
Total susceptible (%)	13/22 (59.1)*	40/48 (83.3)	4.822	0.028
Total protective (%)	12/22 (54.5)*	11/48 (22.9)	6.841	0.008

Susceptible only: patients carry the susceptible HLA haplotypes without protective haplotypes; protective only: patients carry the protective HLA haplotypes without susceptible haplotypes; total susceptible: patients carry at least one of the susceptible HLA haplotypes; total protective: patients carry at least one of the protective HLA haplotypes. *P <0.05 ICI-DM vs T1D.

### HLA-DRB1-DQA1-DQB1 Haplotypes in IFD and IT1D

HLA genotyping was performed in 100% (16/16) of IT1D and 85.7% (6/7) of IFD patients. As shown in **Table 2**, T1D-susceptible DR9 and DRB1* 0405-DQA1*03-DQB1*0401 haplotypes were detected in 25.0% and 8.3% of the IFD patients, respectively. Up to 66.7% (4/6) of IFD patients were heterozygous for reported fulminant type 1 diabetes susceptibility HLA haplotypes (DRB1*0405-DQB1*0401 or DRB1*0901-DQB1*0303).

### Risk HLA-DRB1-DQA1-DQB1 Genotypes in ICI-DM and T1D

The high-risk Chinese T1D genotypes DR3/DR3, DR3/DR9, and DR9/DR9 were not found in ICI-DM patients, whereas the genotypes DR3/DR3, DR3/DR9, and DR9/DR9 were found in 10.4%, 4.2% and 22.9% of T1D patients, respectively (**Table 4**). None of the ICI-DM patients were homozygous for T1D-susceptible HLA Class II haplotypes or heterozygous for two susceptible haplotypes, which was lower than the 45.8% (22/48) in T1D (*P<*0.001). In contrast, 18.2% (4/22) of ICI-DM patients were homozygous for T1D protective HLA Class II haplotypes or heterozygous with two protective haplotypes, higher than the 4.2% (2/48) in T1D (*P*=0.052). None of the ICI-DM and T1D patients had the DR3/DR4 or DR4/DR4 genotype.

**Table 4:** HLA-DRB1-DQA1-DQB1 Susceptible Genotype Frequencies between ICI-DM and T1D.

HLA genotype	ICI-DM (n = 22)	T1D (n=48)
DR3/DR3(%)	0(0.0)	5/48(10.4)
DR3/DR9(%)	0(0.0)	2/48(4.2)
DR9/DR9(%)	0(0.0)	11/48(22.9)
DR3/DR4(%)	0(0.0)	0(0.0)
DR4/DR4(%)	0(0.0)	0(0.0)

## Discussion

ICI-DM is a rare but often life-threatening adverse effect of ICIs. In our cohort, 78.3% of ICI-DM patients presented with diabetic ketoacidosis, 81.8% of patients were treated with anti-PD-1, and all had low or undetectable C-peptide levels, which is consistent with a previous report (21). Compared to classic T1D patients, ICI-DM patients had higher blood glucose and lower HbA1c levels, which may indicate an outbreak onset of diabetes and a short duration of hyperglycemia. As reported, islet autoantibodies were positive in half of ICI-DM patients with a dominance of GADA (51%), 18% for IA-2, 13% for ICA, 26% for IAA and 4% for ZnT8 (21). However, in our cohort, only 2 (8.7%) ICI-DM patients were positive for islet autoantibodies (both had GADA and ICA), which was apparently lower than T1D (66.7%). These findings are consistent with a previous study in Japan, in which only two of ten ICI-DM patients were positive for islet autoantibodies (both had GADA), and no patients were positive for IA-2A, IAA, or Znt8A (5). In contrast, Angeliki (20) et al. found that 40% (10/25) of ICI-DM Caucasian patients were positive for at least one islet autoantibody. It has been reported that the frequencies and levels of islet autoantibodies are related to the HLA-DR-DQ genes (27, 28). In Caucasian populations with classical T1D, GADA positivity and levels were associated with the HLA-DR3 haplotype, whereas IAA and IA-2A positivity were associated with the HLA-DR4 haplotype (29, 30). The relationship between GADA, IA-2A, IAA, and HLA-DR-DQ haplotypes in Chinese populations has not been extensively studied. Wang (31) et al. found that GADA was more prevalent in Chinese T1D patients carrying the DQA1*05-DQB1*0201 or DQA1*03-DQB1*0401 haplotype, whereas IA-2A was more prevalent in patients carrying the DQA1*03-DQB1*0303 haplotype. Peng (32) et al. found that GADA multiepitope positivity was associated with DR3 haplotypes in patients with latent autoimmune diabetes in youth. In Japanese T1D patients, the level of ZnT8A was associated with the HLA-DR4 allele (33). The low frequencies of DR3 and DR4 in our ICI-DM population may in part explain the absence of antibodies to insulin, IA-2A, and ZnT8, but large samples are needed to further confirm our conclusions.

Humoral autoimmunity, including islet-specific autoimmunity, is modulated by HLA-DQ genes. HLA class II molecules expressed on antigen-presenting cells have critical roles in the activation of CD4 T cells and may influence the autoimmune response (34). HLA class II genes are the major genetic predisposition genes for T1D and contribute approximately 50% of the T1D genetic risk (15). However, the effect of HLA susceptibility genes on ICI-DM remains unknown, and most of the data were from Caucasian populations. A previous study showed that there was a predominance of HLA-DR4 (16/21, 76%) in ICI-DM patients (20). In another meta-analysis, HLA haplotypes were analyzed in 51 ICI-DM patients, and 61% were susceptible (mostly DR4), 4% were susceptible and protective, and 16% had protective HLA genotypes (21). In contrast, the data of our study showed that DR9 and DRB1*0405-DQA1*03-DQB1*0401 are the major susceptible haplotypes, which indicated that susceptible HLA-DRB1-DQA1 genes vary among different ethnicities. Compared to T1D, the susceptible DR3 and DR9 haplotypes were less frequent, whereas the protective haplotypes (DRB1*1101-DQA1*05-DQB1*0301 and DRB1*1202-DQA1*0601-DQB1*0301) were more frequent in ICI-DM patients. Furthermore, none of the ICI-DM patients had T1D-associated high-risk genotypes DR3/DR3, DR3/DR9, and DR9/DR9. These findings indicated that a significant discrepancy exists between ICI-DM and T1D in disease susceptibility conferred by HLA genes, which helps to provide additional information about HLA-associated risk for ICI-DM across a more diverse patient population. However, considering the small sample size of our study, additional multicenter studies are needed to evaluate the correlation between HLA susceptibility genes and ICI-DM.

Fulminant type 1 diabetes is particularly prevalent in East Asians but rare in Caucasians (35). However, there is a growing number of FT1D cases in Caucasians with the increased use of ICIs, which indicates the possibility that immune checkpoint molecules play an important role in pathogenesis (11). In our cohort, 30.4% of patients presented with IFD who showed significantly higher plasma glucose levels, lower C-peptide and low HbA1c levels compared to IT1D. To date, there are no available detailed haplotypes in most IFD cases. In our cohort, up to 66.7% (4/6) of IFD patients carried reported fulminant type 1 diabetes susceptibility HLA haplotypes (DRB1*0901-DQB1*0303 or DRB1*0405-DQB1*0401) (36), which suggested that fulminant type 1 diabetes HLA susceptibility alleles may be predictors of IFD.

In conclusion, ICI-DM shares analogous clinical characteristics with T1D, such as acute onset, sudden permanent B-cell failure and insulin dependence. However, the low frequencies of islet autoantibodies and T1D-associated HLA-risk haplotypes indicate that ICI-DM represents a new model distinct from classical T1D. These findings shed light on the pathogenesis underlying ICI-DM.

## Data availability statement

The raw data supporting the conclusions of this article will be made available by the authors, without undue reservation.

## Ethics statement

This study was approved by the Institutional Ethics Committee of the Third Xiangya Hospital (NO. I22169). Written informed consent was obtained from all subjects in this study. All procedures were performed according to the World Medical Association’s Declaration of Helsinki. The patients/participants provided their written informed consent to participate in this study.

## Author contributions

Y-CL performed experimental work and paper drafting. HL, S-LZ and KC collected the clinical data and management of patients. PJ designed the research study, paper revision and fund support. All authors contributed to the article and approved the submitted version.

## References

[1] Ribas A. Tumor immunotherapy directed at PD-1. N Engl J Med (2012) 366:2517–19. doi: 10.1056/NEJMe1205943 22658126

[2] Quandt Z Young A Anderson M. Immune checkpoint inhibitor diabetes mellitus: a novel form of autoimmune diabetes. Clin Exp Immunol (2020) 200:131–40. doi: 10.1111/cei.13424 PMC7160652 32027018

[3] Postow MA Sidlow R Hellmann MD. Immune-related adverse events associated with immune checkpoint blockade. N Engl J Med (2018) 378:158–68. doi: 10.1056/NEJMra1703481 29320654

[4] Chang LS Barroso-Sousa R Tolaney SM Hodi FS Kaiser UB Min L. Endocrine toxicity of cancer immunotherapy targeting immune checkpoints. Endocr Rev (2019) 40:17–65. doi: 10.1210/er.2018-00006 30184160 PMC6270990

[5] Tsang VHM McGrath RT Clifton-Bligh RJ Scolyer RA Jakrot V Guminski AD. Checkpoint inhibitor–associated autoimmune diabetes is distinct from type 1 diabetes. J Clin Endocrinol Metab (2019) 104:5499–506. doi: 10.1210/jc.2019-00423 31265074

[6] Kotwal A Haddox C Block M Kudva YC. Immune checkpoint inhibitors: an emerging cause of insulin-dependent diabetes. BMJ Open Diabetes Res Care (2019) 7:e591. doi: 10.1136/bmjdrc-2018-000591 PMC6398813 30899528

[7] Barroso-Sousa R Barry WT Garrido-Castro AC Hodi FS Min L Krop IE. Incidence of endocrine dysfunction following the use of different immune checkpoint inhibitor regimens. JAMA Oncol (2018) 4:173. doi: 10.1001/jamaoncol.2017.3064 28973656 PMC5838579

[8] Liu J Zhou H Zhang Y Fang W Yang Y Huang Y. Reporting of immune checkpoint inhibitor therapy-associated diabetes, 2015-2019. Diabetes Care (2020) 43:e79–80. doi: 10.2337/dc20-0459 PMC7305010 32393586

[9] Usui Y Udagawa H Matsumoto S Imai K Ohashi K Ishibashi M. Association of serum anti-GAD antibody and HLA haplotypes with type 1 diabetes mellitus triggered by nivolumab in patients with non-small cell lung cancer. J Thorac Oncol (2017) 12:e41–43. doi: 10.1016/j.jtho.2016.12.015 28017788

[10] Qiu J Luo S Yin W Guo K Xiang Y Li X. Characterization of immune checkpoint inhibitor-associated fulminant type 1 diabetes associated with autoantibody status and ethnic origin. Front Immunol (2022) 13:968798. doi: 10.3389/fimmu.2022.968798 36451831 PMC9702060

[11] Gaudy C Clevy C Monestier S Dubois N Preau Y Mallet S. Anti-PD1 pembrolizumab can induce exceptional fulminant type 1 diabetes. Diabetes Care (2015) 38:e182–83. doi: 10.2337/dc15-1331 26310693

[12] Telo GH Carvalhal GF Cauduro C Webber VS Barrios CH Fay AP. Fulminant type 1 diabetes caused by dual immune checkpoint blockade in metastatic renal cell carcinoma. Ann Oncol (2017) 28:191–92. doi: 10.1093/annonc/mdw447 28043983

[13] Saito D Oikawa Y Yano Y Ikegami Y Satomura A Isshiki M. Detailed time course of decline in serum c-peptide levels in anti-programmed cell death-1 therapy-induced fulminant type 1 diabetes. Diabetes Care (2019) 42:e40–41. doi: 10.2337/dc18-1673 30659072

[14] Baden MY Imagawa A Abiru N Awata T Ikegami H Uchigata Y. Characteristics and clinical course of type 1 diabetes mellitus related to anti-programmed cell death-1 therapy. Diabetol Int (2019) 10:58–66. doi: 10.1007/s13340-018-0362-2 30800564 PMC6357237

[15] Noble JA Valdes AM Cook M Klitz W Thomson G Erlich HA. The role of HLA class II genes in insulin-dependent diabetes mellitus: molecular analysis of 180 Caucasian, multiplex families. Am J Hum Genet (1996) 59:1134–48. PMC1914851 8900244

[16] Erlich H Valdes AM Noble J Carlson JA Varney M Concannon P. HLA DR-DQ haplotypes and genotypes and type 1 diabetes risk: analysis of the type 1 diabetes genetics consortium families. Diabetes (2008) 57:1084–92. doi: 10.2337/db07-1331 PMC4103420 18252895

[17] Park Y She JX Wang CY Lee H Babu S Erlich HA. Common susceptibility and transmission pattern of human leukocyte antigen DRB1-DQB1 haplotypes to Korean and Caucasian patients with type 1 diabetes. J Clin Endocrinol Metab (2000) 85:4538–42. doi: 10.1210/jcem.85.12.7024 11134105

[18] Kawasaki E Matsuura N Eguchi K. Type 1 diabetes in Japan. Diabetologia (2006) 49:828–36. doi: 10.1007/s00125-006-0213-8 16568259

[19] Luo S Lin J Xie Z Xiang Y Zheng P Huang G. HLA genetic discrepancy between latent autoimmune diabetes in adults and type 1 diabetes: LADA China study no. 6. J Clin Endocrinol Metab (2016) 101:1693–700. doi: 10.1210/jc.2015-3771 26866570

[20] Stamatouli AM Quandt Z Perdigoto AL Clark PL Kluger H Weiss SA. Collateral damage: insulin-dependent diabetes induced with checkpoint inhibitors. Diabetes (2018) 67:1471–80. doi: 10.2337/dbi18-0002 PMC6054443 29937434

[21] de Filette J Pen JJ Decoster L Vissers T Bravenboer B van der Auwera BJ. Immune checkpoint inhibitors and type 1 diabetes mellitus: a case report and systematic review. Eur J Endocrinol (2019) 181:363–74. doi: 10.1530/EJE-19-0291 PMC6709545 31330498

[22] Marchand L Thivolet A Dalle S Chikh K Reffet S Vouillarmet J. Diabetes mellitus induced by PD-1 and PD-L1 inhibitors: description of pancreatic endocrine and exocrine phenotype. Acta Diabetol (2019) 56:441–48. doi: 10.1007/s00592-018-1234-8 30284618

[23] Imagawa A Hanafusa T Awata T Ikegami H Uchigata Y Osawa H. Report of the committee of the Japan diabetes society on the research of fulminant and acute-onset type 1 diabetes mellitus: new diagnostic criteria of fulminant type 1 diabetes mellitus (2012). J Diabetes Investig (2012) 3:536–39. doi: 10.1111/jdi.12024 PMC4015434 24843620

[24] Nan X Li X Xiang Y Yan X Zhou H Tang X. Screening strategy for islet autoantibodies in diabetes patients of different ages. Diabetes Technol Ther (2022) 24:212–19. doi: 10.1089/dia.2021.0177 34704825

[25] Wang Z Li X Yan X Zhang XL Liu N Li MW. [A novel electrochemiluminescence method for detecting insulin autoantibody]. Zhonghua Yi Xue Za Zhi (2020) 100:424–29. doi: 10.3760/cma.j.issn.0376-2491.2020.06.006 32146764

[26] Xia Y Li X Huang G Lin J Luo S Xie Z. The association of HLA-DP loci with autoimmune diabetes in Chinese. Diabetes Res Clin Pract (2021) 173:108582. doi: 10.1016/j.diabres.2020.108582 33307130

[27] Krischer JP Lynch KF Schatz DA Ilonen J Lernmark A Hagopian WA. The 6 year incidence of diabetes-associated autoantibodies in genetically at-risk children: the TEDDY study. Diabetologia (2015) 58:980–87. doi: 10.1007/s00125-015-3514-y PMC4393776 25660258

[28] Mayr A Schlosser M Grober N Kenk H Ziegler AG Bonifacio E. GAD autoantibody affinity and epitope specificity identify distinct immunization profiles in children at risk for type 1 diabetes. Diabetes (2007) 56:1527–33. doi: 10.2337/db06-1715 17325256

[29] Knip M Kukko M Kulmala P Veijola R Simell O Akerblom HK. Humoral beta-cell autoimmunity in relation to HLA-defined disease susceptibility in preclinical and clinical type 1 diabetes. Am J Med Genet (2002) 115:48–54. doi: 10.1002/ajmg.10343 12116176

[30] Triolo TM Pyle L Broncucia H Armstrong T Yu L Gottlieb PA. Association of high-affinity autoantibodies with type 1 diabetes high-risk HLA haplotypes. J Clin Endocrinol Metab (2022) 107:e1510–17. doi: 10.1210/clinem/dgab853 PMC8947772 34850014

[31] Wang JP Zhou ZG Lin J Huang G Zhang C Yang L. Islet autoantibodies are associated with HLA-DQ genotypes in han Chinese patients with type 1 diabetes and their relatives. Tissue Antigens (2007) 70:369–75. doi: 10.1111/j.1399-0039.2007.00916.x 17919266

[32] Peng Y Li X Xiang Y Yan X Zhou H Tang X. GAD65 antibody epitopes and genetic background in latent autoimmune diabetes in youth (LADY). Front Immunol (2022) 13:836952. doi: 10.3389/fimmu.2022.836952 35392100 PMC8982141

[33] Kawasaki E Nakamura K Kuriya G Satoh T Kobayashi M Kuwahara H. Differences in the humoral autoreactivity to zinc transporter 8 between childhood- and adult-onset type 1 diabetes in Japanese patients. Clin Immunol (2011) 138:146–53. doi: 10.1016/j.clim.2010.10.007 21067978

[34] Nishimura Y Oiso M Fujisao S Kanai T Kira J Chen YZ. Peptide-based molecular analyses of HLA class II-associated susceptibility to autoimmune diseases. Int Rev Immunol (1998) 17:229–62. doi: 10.3109/08830189809054404 10036633

[35] Luo S Zhang Z Li X Yang L Lin J Yan X. Fulminant type 1 diabetes: a collaborative clinical cases investigation in China. Acta Diabetol (2013) 50:53–9. doi: 10.1007/s00592-011-0362-1 22193926

[36] Kochupurakkal NM Kruger AJ Tripathi S Zhu B Adams LT Rainbow DB. Blockade of the programmed death-1 (PD1) pathway undermines potent genetic protection from type 1 diabetes. PloS One (2014) 9:e89561. doi: 10.1371/journal.pone.0089561 24586872 PMC3938467

